# Decoding Biosynthetic Pathways in Plants by Pulse-Chase Strategies Using ^13^CO_2_ as a Universal Tracer †

**DOI:** 10.3390/metabo6030021

**Published:** 2016-07-14

**Authors:** Adelbert Bacher, Fan Chen, Wolfgang Eisenreich

**Affiliations:** Lehrstuhl für Biochemie, Technische Universität München, 85748 Garching, Germany; adelbert.bacher@t-online.de (A.B.); fan.chen@mytum.de (F.C.)

**Keywords:** biosynthesis, metabolism, isotopologue profiling, ^13^CO_2_, flux analysis

## Abstract

^13^CO_2_ pulse-chase experiments monitored by high-resolution NMR spectroscopy and mass spectrometry can provide ^13^C-isotopologue compositions in biosynthetic products. Experiments with a variety of plant species have documented that the isotopologue profiles generated with ^13^CO_2_ pulse-chase labeling are directly comparable to those that can be generated by the application of [U-^13^C_6_]glucose to aseptically growing plants. However, the application of the ^13^CO_2_ labeling technology is not subject to the experimental limitations that one has to take into account for experiments with [U-^13^C_6_]glucose and can be applied to plants growing under physiological conditions, even in the field. In practical terms, the results of biosynthetic studies with ^13^CO_2_ consist of the detection of pairs, triples and occasionally quadruples of ^13^C atoms that have been jointly contributed to the target metabolite, at an abundance that is well above the stochastic occurrence of such multiples. Notably, the connectivities of jointly transferred ^13^C multiples can have undergone modification by skeletal rearrangements that can be diagnosed from the isotopologue data. As shown by the examples presented in this review article, the approach turns out to be powerful in decoding the carbon topology of even complex biosynthetic pathways.

## 1. Introduction

Isotope incorporation analysis is a time-honored, major tool for the dissection of biosynthetic and metabolic processes. Labeling experiments using ^2^H-isotopes were introduced in the 1930s when heavy water, ^2^H_2_O, became available [1]. Until about 1960, isotope use in biochemistry was then mostly dominated by the radioisotopes ^3^H and ^14^C [2,3]. Later, a variety of factors have been conducive to a progressive shift to the use of stable isotopes. Notably, stable isotopes involve no health hazards and are exempt from safety regulations. Moreover, dramatic progress in NMR and mass spectrometry instrumentation has greatly enhanced the sensitivity of stable isotope detection [4,5].

The major elemental components of organic matter, i.e., hydrogen, carbon, nitrogen and oxygen, are all present in nature as mixtures of stable isotopes. In natural organic matter, these isotopes show an almost random distribution. Small deviations from randomness arise by isotope fractionation inherent in chemical (including biochemical) reactions [6], but the non-random effects are small, and sensitive techniques are required for their assessment; these aspects are not addressed in the present study.

Isotope tracer techniques designed to analyze biosynthetic and other metabolic processes invariably start with an artificially-induced perturbation of natural isotope distribution. In principle, a perturbation can be caused by the introduction of any chemical with an isotope distribution at variance from the average isotope composition of natural organic matter. More specifically, the perturbing agent could be universally isotope enriched or enriched at single or multiple discrete sites. Crucially, however, it needs to be able to access the metabolic network of the study organism.

Selecting an appropriate tracer, with regard to the structure and labeling pattern of isotope-labeled tracers, depends on numerous boundary conditions, including, but not limited to (i) commercial or synthetic availability; (ii) uptake by the target organism and (iii) the availability (or absence) of a strong working hypothesis suggesting a biosynthetic relationship of the tracer and target product. A brief discussion of the network theory of intermediary metabolism is required in order to gain a rational basis for the choice of tracers under a wide variety of conditions.

## 2. Metabolic Networks

The numbers of open reading frames specified by different genomes are now known to range from a few hundred (in the case of certain microorganisms) to tens of thousands [7]. Although not all translation products serve as enzymes, the numbers of enzymes and, hence, putative enzyme products are likewise in the range of hundreds to many thousands. By interpreting metabolites as nodes that can be connected by enzymes catalyzing the conversion of certain metabolites into other metabolites, the metabolome of any given organism can be described as a network whose complexity depends on the number of enzymes specified by the organism’s genome and by the specific connectivities of each respective node.

**Figure 1:**
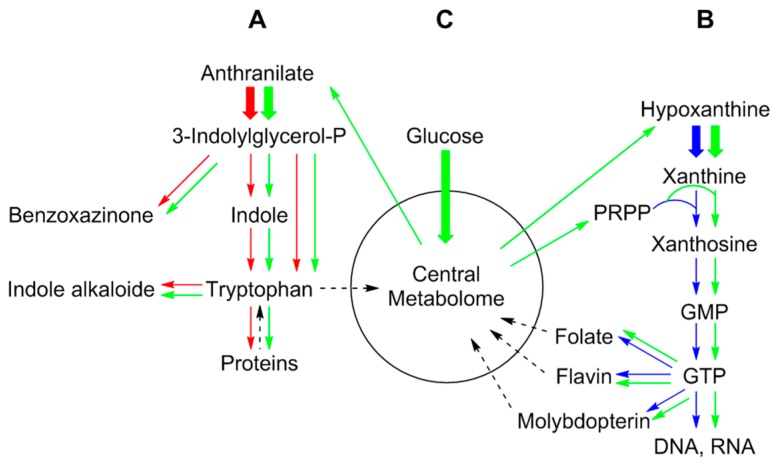
Principal strategies for in vivo labeling experiments. A: Incorporation experiment with a metabolite (i.e., anthranilate in the example) biogenetically close to the target metabolite (i.e., indole alkaloids or benzoxazinones in the example). The label can be distributed over a limited part of the metabolic network shown by red arrows. B: Incorporation experiment with a metabolic precursor (i.e., hypoxanthine in the example) for a wider range of products (folates, flavines, pterins, DNA/RNA) shown by blue arrows. C: Incorporation experiment with a general precursor (i.e., glucose in the example) where the label is distributed via the central metabolome of the organism shown by green arrows. PRPP, 5-phosphoribosyl diphosphate.

Unsurprisingly, certain parts of metabolic networks are more closely interconnected than other parts. Notably, central intermediary metabolism (cf. the central metabolome in Figure 1), e.g., dealing with the interconversion of carbohydrates or carboxylic acids, is typically characterized by dense connectivity [8,9]. On the other hand, biosynthetic pathways conducive to structurally complex primary metabolites (such as aliphatic and aromatic amino acids, vitamins) and secondary metabolites (e.g., antibiotics, alkaloids) are more in the nature of “one-way roads” where nodes frequently have only two connections (to the direct precursor and the consecutive intermediary product, respectively).

The introduction of a node compound, in isotope-labeled form, into the metabolome of a given organism converts that node into a source for perturbation of the near-equilibrium distribution of isotopes in natural matter. Depending on the number of connections of the given node, the potential division of the global network into modular sections [10,11] and the interconnections between these sections, the perturbation can spread into many or few directions where other nodes could serve as novel sources of the tracer isotope.

As a simple case, consider the feeding of ^13^C-labeled anthranilic acid to a microorganism that is capable of tryptophan biosynthesis [12] (Figure 1). The labeled tracer will be incorporated, via 3-indolylglycerol phosphate and indole, into the benzenoid ring of tryptophan, into proteins and potentially into all secondary products downstream from tryptophan, such as certain alkaloids; however, the central metabolome can only be accessed after catabolic degradation of the proffered precursor or one of its downstream products.

As an example for a more general, but still limited mode of spreading, let us consider the introduction of an isotope-labeled purine base such as hypoxanthine (Figure 1). The perturbant will be able to access the complex network of purine nucleotides by the transfer of a ribose phosphate moiety, thus converting it into IMP, which will then serve as a secondary perturbation source [13]. From that secondary source, the label (and, hence, the perturbation) will be able to spread to a variety of sinks, including the entire purine nucleotide and deoxynucleotide network, and the realm of nucleic acids, but also the realm of purine-derived primary and secondary metabolites such as coenzymes (e.g., flavocoenzymes, folic acid derivatives, the molybdopterin cofactor family), some special tRNA bases, purine-derived antibiotics, to name only the most important [14,15,16]. Nevertheless, the spread of the perturbation will be far from universal, since the label will need a relatively long time to access the central carbohydrate pools (in fact, this will only be possible after catabolic disruption of the purine ring system that would be conducive to very simple molecules, such as formate and CO_2_).

A totally different situation arises when a major nutrient, such as glucose, is proffered in isotope-labeled form (Figure 1). After penetration into the cytoplasm, the tracer will immediately start to be diverted to all metabolic compartments, and the isotope label will spread out throughout the metabolic network (with intracellular glucose or its phosphoric acid ester, respectively, serving as a source, virtually all nodes will operate as sinks within a short time period). Hence, the net transfer of isotope to all nodes in the network fails to provide meaningful information about metabolic processes, unless we make use of the fact that isotope atoms do not travel the metabolic network alone, but as tightly (covalently) linked associations of atoms. In other words, the carbon skeletons of metabolites are pieced together as a patchwork assembled from precursor metabolites or their respective fragments by the catalytic action of enzymes.

As a specific (and fairly typical) example, consider the application of universally ^13^C-labeled glucose ([U-^13^C_6_]glucose) to a biological target. Following its incorporation, it may access one or more catabolic pathways resulting in fragmentation, affording compounds carrying one (in the case of catabolite ^13^CO_2_), two or more ^13^C atoms, which are contributed to the inventory of central intermediary metabolites. Henceforth, anabolic processes will start from mixtures of unlabeled and labeled intermediates of central intermediary metabolism (unlabeled intermediates from the metabolic inventory that had been established prior to the addition of the ^13^C-labeled tracer, labeled intermediates obtained by catabolism of the ^13^C-labeled tracer and their reactions with components of the unlabeled metabolic inventory). The products resulting from anabolic processes will then appear as mosaics with contributions by ^13^C-labeled, as well as unlabeled building blocks. Following the isolation of individual metabolites from the experimental system, contiguous groups of ^13^C atoms in the metabolites under study can be diagnosed by NMR spectroscopy or by the combined application of NMR and MS techniques.

**Figure 2:**
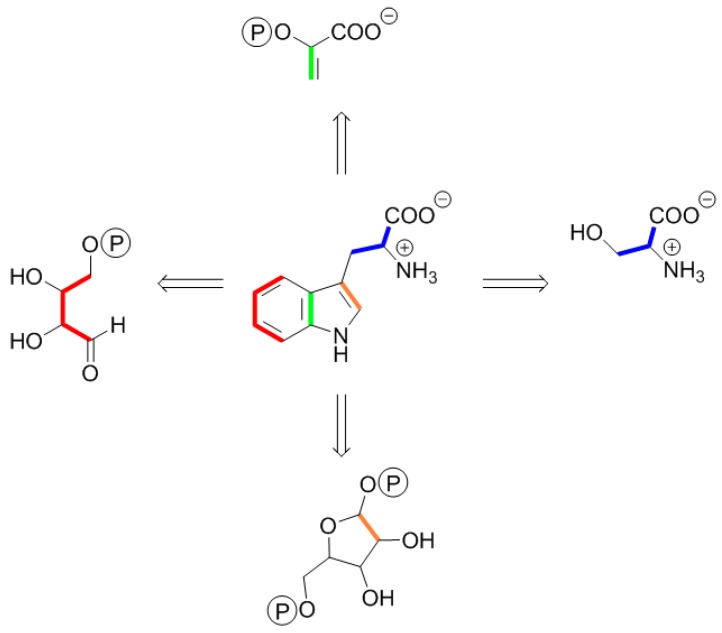
Retrobiosynthetic dissection of tryptophan. The aromatic amino acid is formed via the shikimate pathway using erythrose 4-phosphate (red labels), phosphoenol pyruvate (green labels), serine (blue labels) and phosphoribosyl diphosphate (orange labels) as building blocks. On the basis of the detected labeling pattern of tryptophan, the labeling patterns of the precursors are reconstructed as shown by “retro” arrows. The colors represent biosynthetically-equivalent modules.

The interpretation of tracer studies using multiply-labeled central metabolic intermediates typically involves the comparison of the labeling patterns of numerous metabolites from a given experiment. Importantly, the available information on the biosynthetic pathways of primary metabolites can be used for the reconstruction of the labeling patterns of central metabolic pools. For example, since tryptophan is exclusively biosynthesized via the shikimate pathway, the labeling pattern of tryptophan from a given labeling experiment allows the unequivocal reconstruction (retrodiction) of the labeling pattern of erythrose 4-phosphate (Figure 2). Moreover, the labeling pattern of tryptophan also informs about the labeling of carbon atoms 1 and 2 of the pentose phosphate pool and of carbon atoms 2 and 3 of phosphoenol pyruvate.

The synopsis of retrodictive information from amino acids affords a detailed description of the labeling patterns of central metabolic intermediates (Table 1) that can then also be used as the basis for a pattern recognition approach with the aim to identify the building blocks in the biosynthesis of a metabolite under study.

## 3. Technical Hurdles for Tracer Studies in Plants

Whereas the presented arguments apply, in principle, to studies in all biological kingdoms, specific problems arise with the application of isotope-labeled tracers to plants. With the exception of saprophytic and parasitic species, plants generate their biomass exclusively from CO_2_. Pilot experiments with aseptically grown tobacco plants have provided proof of principle for feeding of ^13^C-labeled glucose via the root system [17]. However, the availability of aseptically-growing plant specimens is the exception rather than the norm. Thus, conducting tracer experiments with unperturbed plants is not easily feasible.

Work-arounds are possible and have been successfully used in numerous cases. Plant cell cultures or plant organ cultures (e.g., embryos or root cultures) growing in vitro enable experiments that are technically similar to work with microorganism [18,19,20]. Again, the availability of these systems for a given species will be the exception rather than the norm. Moreover, plant cells or organs growing in vitro may or may not be producing specific plant metabolites in quantities that are sufficient for analysis. Nevertheless, important problems in plant biochemistry and physiology could be solved with that approach.

Experiments can also be conducted with cut plant segments that are immersed into nutrient solutions containing a tracer compound. However, cut segments are less metabolically prolific than whole plants; moreover, their condition is far from being physiological, is subject to progressive decline with time and, finally, may reflect metabolic pathways and fluxes triggered by the wounding of the plant and being absent in the intact plant under standard conditions. In addition, as with cultured plant cells, the metabolic productivity of cut segments may be less than that of intact plants.

The single direct carbon source for most plants growing under natural conditions (with the exception of saprophytes and parasites) is of course CO_2_. This review article describes examples for tracer experiments with ^13^CO_2_ directed at the elucidation of biosynthetic pathways of plant secondary metabolites. The extensive use of ^13^CO_2_ for a variety of research purposes in general plant physiology (for recent examples, see [21,22,23,24,25,26,27]) is not covered in this review. It is important to note that work on biosynthesis depends technically on high resolution NMR of metabolites that have been isolated in pure form. Due to the limited resolution and sensitivity of NMR spectroscopy, biosynthetic studies using ^13^CO_2_ as a tracer have only been rarely used in the past century [28]. In contrast to ^13^CO_2_-based biosynthetic studies at high NMR resolution, general plant physiology analyses with ^13^CO_2_ can also use solid state NMR techniques enabling the analysis of intact plant tissue, as initiated by work from the research group of Schaefer and others (for specific examples, cf. [29,30,31,32]).

## 4. Biochemistry of ^13^CO_2_ Pulse-Chase Experiments

Under conditions of optimal lighting, plants make adjustments for maximum utilization of the incoming light for photosynthesis. When proffered exclusively with ^13^CO_2_ as the carbon source, photosynthesis will then generate ^13^C-labeled carbohydrates that can be partially stored away as starch. In an ideal (but hypothetical) experiment, the photosynthetic products would approach universal labeling at a level equivalent to the ^13^C abundance of the feeding CO_2_, above 99%. However, since growing plants are endowed with an inventory of unlabeled metabolites and sugars (already present in the plants prior to the labeling experiment), the ^13^C enrichments of these products will be lower (typically 0.2%–5% ^13^C excess in the isolated target compounds) than the enrichment of the proffered ^13^CO_2_ (i.e., 99% ^13^C excess) during the short pulse period of the labeling experiment (typically 1–10 h).

When the plants are returned to normal atmosphere for a predetermined chase phase (typically 1–10 days), two different processes will play out: (i) during light periods, the plants under study will now produce new carbohydrates from CO_2_ with natural ^13^C abundance of about 1.1%; (ii) during dark periods, the plants will use stored metabolites as sources of energy and metabolism and will thus mobilize ^13^C-labeled metabolites from the pulse period; moreover, they will utilize unlabeled storage material that had been deposited prior the pulse period (quasi-“fossil” storage material), as well as low molecular weight and high molecular weight material generated during the chase period from CO_2_ with natural (i.e., low) ^13^C abundance.

The first committed step of carbon fixation in plants is catalyzed by ribulose bisphosphate carboxylase (RUBISCO), which generates two equivalents of 3-phosphoglycerate from one equivalent each of ribulose bisphosphate and CO_2_ [33]. 3-Phosphoglycerate can be converted into ribulose bisphosphate via the Calvin cycle under regeneration of 3-phosphoglycerate, which serves as the primary CO_2_ acceptor in the RUBISCO reaction. When a plant is grown in a synthetic atmosphere containing ^13^CO_2_, the carbon isotope will therefore access the inventory of Calvin cycle intermediates, resulting in a progressive wash-in of ^13^C (and concomitant wash-out of ^12^C) affecting the 3-phosphoglyerate pool and the whole metabolic network downstream from 3-phosphoglycerate. Thus, the average ^13^C abundance of the soluble carbohydrate inventory should asymptotically approach the ^13^C abundance of the proffered CO_2_ by way of monotonous progression.

In a typical pulse/chase experiment, the ^13^CO_2_-treated plant is subsequently returned to normal atmosphere (with different terminology, the ^13^CO_2_ phase can be interpreted as the perturbation phase and the consecutive phase in normal atmosphere as the relaxation period in a perturbation/relaxation experiment). In the chase period (relaxation period), the plant will now produce additional photosynthate derived from natural CO_2_ comprising predominantly ^12^C; moreover, unlabeled carbohydrates may also become available by the catabolism of storage polymers that had already been present prior to the ^13^CO_2_ pulse phase. For the biosynthesis of primary and secondary metabolites, the plant can now retrieve material from all currently available pools of photosynthate (i.e., from unlabeled pre-pulse storage material, from labeled pulse-phase material and from unlabeled chase-phase material). Notably, carbohydrate molecules that had been formed in different periods can jointly undergo metabolic recycling.

As a first approximation, the plant will be operating under conditions that are similar to a plant that is growing aseptically with an exogenous supply of [U-^13^C_6_]glucose. In fact, there is detailed experimental evidence for this hypothesis [17,34] that will be presented below. However, in order to present that evidence, it is essential to introduce some technical details concerning the spectroscopic analysis of the metabolites. Readers who are familiar with the spectroscopic analysis of isotopologue mixtures are advised to skip the following paragraphs.

## 5. Terminology of ^13^C Isotopologue Mixtures

According to the rules of the International Union of Pure and Applied Chemistry (IUPAC), isomers having the same number of each isotopic atom, but differing in their positions are termed isotopomers. In contrast, molecular entities that differ more generally in their isotopic compositions are noted isotopologues. Since the latter term involves the full range of molecules that are subjected to NMR or MS analysis in the ^13^C-labeling experiments, we prefer the isotopologue notation. IUPAC rules for the accurate description of isotopologues use square brackets to indicate the position, type and number of isotopic nuclei in a given isotopologue. For example, [1-^13^C_1_]glucose carries a single ^13^C label in position 1; [2,3-^13^C_2_]glucose carries ^13^C labels in positions 2 and 3; and all carbon atoms are ^13^C in [U-^13^C_6_]glucose. Working with ^13^CO_2_ as the tracer, one has to deal with complex sets of multiple isotopologues. Hence, we found it necessary to introduce a shorthand notation that is easily accessible to being handled by set theory. Briefly, the isotopologue status of a compound comprising n carbon atoms is rendered as a binary number with n positions, where each digit describes the status of a specific carbon atom. ^12^C is rendered as 0 (zero); ^13^C is rendered as 1 (one); and x serves as a wild card designating a molecular position with unknown isotope status. For example, [1-^13^C_1_]-, [2,3-^13^C_2_]- and [U-^13^C_6_]glucose are rendered as {100000}, {011000} and {111111}, respectively. Isotope mixtures are written as sets of binary numbers; hence {100000, 011000, 111111} designates the mixture of the three isotopologues mentioned above. {11xxxx}Glucose designates a mixture of isotopologues with ^13^C in positions 1 and 2, but with the carbon isotope status of the remaining carbon atoms being not declared.

## 6. A Short Primer to ^13^C-NMR Spectra of Complex Isotopologues and Isotopologue Mixtures

**Figure 3:**
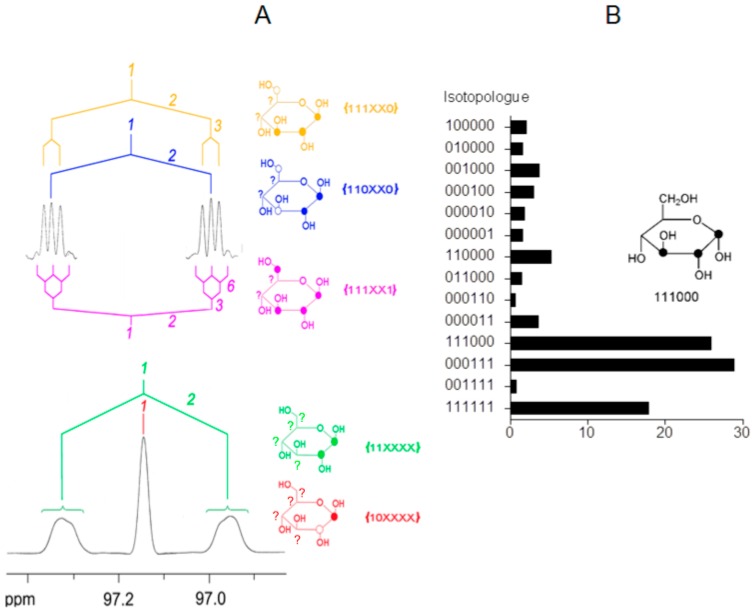
^13^C-NMR-based isotopologue profiling of complex isotopologue mixtures. (**A**) ^13^C-NMR signature of C-1 in glucose isolated from starch of maize kernels grown on agar containing [U-^13^C_6_]glucose and unlabeled glucose [36]. The same ^13^C-NMR signal when processed by multiplication with an exponential function (bottom) or a Gaussian function (top). Signals due to scalar couplings between ^13^C-atoms in the same molecule are indicated. The coupling partners are indicated by italic numbers. Colors indicate the corresponding x-groups with 1 = ^13^C, 0 = ^12^C and X = unknown. Filled or open circles in the structures indicate ^13^C- or ^12^C-atoms, respectively. (**B**) ^13^C-Isotopologue abundances mol% in the ^13^C-enriched glucose) by numerical deconvolution of the detected x-group abundances. One of the major isotopologues in the biosynthetic mixture, i.e., [1,2,3-^13^C_3_]glucose due to glycolytic cycling of the proffered [U-^13^C_6_]glucose, is displayed [36].

Generally, the detection and quantification of ^13^C isotopologue mixtures requires high-resolution NMR instruments (>400 MHz ^1^H-frequency), preferably equipped with a cryo-probe head enabling ^13^C-detection at high sensitivity (i.e., with the inner coil tuned to ^13^C). In ^13^C-NMR spectra of organic matter with natural ^13^C abundance (i.e., about 1.1% ^13^C), signals appear as singlets (since the ^13^C-^13^C coupling satellites typically disappear in the noise at 1.1% natural ^13^C abundance). The probability for directly adjacent ^13^C pairs is in the range of 100 ppm, and the probability of directly connected ^13^C triples is in the range of 1 ppm; thus, the general probability of directly-connected ^13^C pairs is low, and the probability of larger directly =connected ^13^C ensembles is even lower. However, the abundance of molecules carrying ensembles of covalently connected ensembles of ^13^C atoms can be dramatically enhanced by pulse labeling of intact plants with ^13^CO_2_. A consecutive chase period in air with natural ^13^CO_2_ abundance results in the formation of metabolites containing multiply-labeled molecular species in the single-digit % range, i.e., with an abundance of multiply ^13^C-labeled molecular species that is orders of magnitude above their occurrence in natural organic material. ^13^C spectra of samples derived from that type of experiment described in more detail below typically show much higher complexity. When ensembles of two or more ^13^C atoms are connected by one to four covalent bonds, their signals appear split into multiplets that can be rather complex. The ^13^C-^13^C coupling constants relating directly-connected carbon atoms are in the range of 30–70 Hz. The connectivity of ^13^C atoms via 2–4 bonds can show up with coupling constants up to 10 Hz. The subcomponents of a given multiplet can be directly related to sets of certain isotopologues (Figure 3).

The binary notation introduced was specifically designed for the quantitative analysis of isotopologue mixtures by ^13^C-NMR spectroscopy. The information gleaned from specific multiplets can be expressed in the format of x-groups (sets of isotopologues where the isotopologue status of certain molecular positions is unknown and is rendered as x). The comprehensive analysis of a given spectrum affords quantitative information on certain x-group sets. From these, the abundance of individual isotopologues can be extracted by numerical deconvolution. The technical details have been described earlier, and readers are directed to those papers for specifics [17,34,35].

## 7. Similar Labeling Patterns of Metabolic Glucose in Tobacco Plants Exposed to [U-^13^C_6_]glucose or ^13^CO_2_

**Figure 4:**
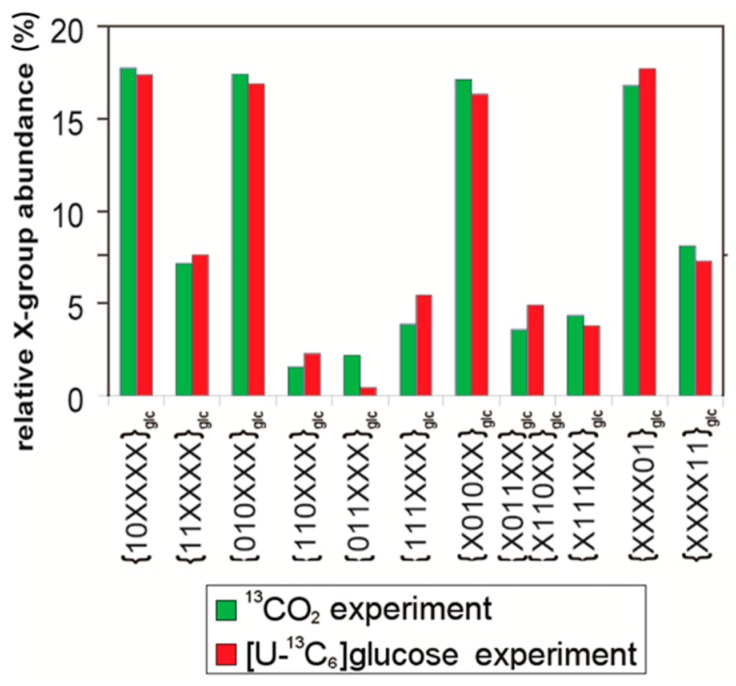
Comparison of x-group profiles of leaf glucose (relative fractions in ^13^C-enriched samples) harvested from tobacco plants (i) after pulse-chase with ^13^CO_2_ (in green) and (ii) after growth with [U-^13^C_6_]glucose supplied via the root system of a plant growing on a matrix of sterile agar (in red) [34].

Figure 4 compares the x-group profiles of glucose harvested from tobacco plants (i) after pulse-chase with ^13^CO_2_ and (ii) after growth with [U-^13^C_6_]glucose supplied via the root system of a plant growing on a matrix of sterile agar [34]. The patterns show remarkable similarity, which is not surprising in light of the foregoing considerations. With this in mind, it appears reasonable to use the interpretational approach that has been developed in work with [U-^13^C_6_]glucose as tool for the interpretation of ^13^CO_2_ labeling work.

The established technique for the interpretation of biosynthetic and metabolomics data from the relatively large body of evidence generated with [U-^13^C_6_]glucose has been reviewed in some detail [37]. Briefly, the carbon skeletons of primary and secondary metabolites are mosaics that are assembled from building blocks derived from the inventory of carbohydrates and carbonic acids of central intermediary metabolites. The initial metabolization of proffered [U-^13^C_6_]glucose in plants can proceed via glycolysis and the pentose phosphate cycle (which are both participants in the more complex Calvin cycle). These processes involve the breaking of ^13^C-^13^C bonds and generate novel molecular entities comprising fewer than six ^13^C atoms. When these molecular entities are used as building blocks in an environment with an abundance of unlabeled molecules (from biomass that was present prior to the introduction of the ^13^C-labeled glucose), novel carbon-carbon bonds will be formed, which implicate covalent linkage between fragments derived from ^13^C-labeled material, as well as from unlabeled material. Thus, the newly-generated molecules are mosaics with labeled and unlabeled domains, depending on the specific origin of each respective building block.

The biosynthetic pathways conducive to certain primary metabolites in plants are known in detail. Biosynthetic amino acids can be isolated conveniently after hydrolysis of biomass, and their isotopologue patterns can be determined by NMR. Mass spectroscopy can be used for further confirmation when required. On this basis, the labeling patterns of many central intermediary metabolites can be reconstructed on the basis of the labeling profiles of amino acids [37]. For example, aromatic amino acids are exclusively formed via the shikimate pathway. Specifically, the carbon skeleton of tryptophan arises from erythrose phosphate, phosphoenol pyruvate, ribose phosphate and serine (cf. Figure 2). The labeling pattern of erythrose 4-phosphate and serine used as the source material for biosynthetic tryptophan can thus be easily deduced in their entirety from the labeling pattern of tryptophan. On the other hand, since only two respective carbon atoms are contributed to the amino acid from phosphoenol pyruvate and ribulose phosphate, the information accessible from the tryptophan labeling pattern is restricted to the label distribution in carbon atoms 1 and 2 of ribose phosphate or, more generally speaking, of the pentose phosphate pool, and carbon atoms 2 and 3 of phosphoenol pyruvate; still, this is useful information in the context of additional information gleaned from the labeling patterns of other amino acids and nucleic acid building blocks (the latter being of course ideally suited to diagnose the labeling of the pentose phosphate pool).

The labeling patterns of secondary metabolites can also be determined by NMR and by supplementary MS analysis as required. Since the carbon skeletons of secondary metabolites are assembled from participants of central intermediary metabolites (e.g., carbohydrates and carbonic acids) and/or from more complex primary metabolites, such as amino acids and nucleic acid building blocks, it stands to reason that the labeling patterns of the primary building blocks should be reflected in the labeling patterns of more complex metabolites (a special problem arises when the mosaic pattern of a complex metabolite is somehow scrambled by one or several structural rearrangements; however, the reconstruction of the primary building blocks is still possible and will be illustrated below in connection with isoprenoid biosynthesis).

## 8. Comparison of Labeling Patterns of Plant Secondary Products as Observed in Experiments with [U-^13^C_6_]glucose or ^13^CO_2_

### 8.1. Nicotine

**Figure 5:**
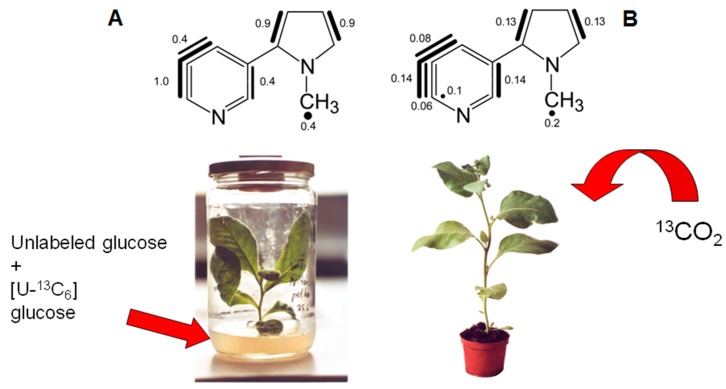
Experimental settings for labeling experiments for the analysis of nicotine biosynthesis in tobacco plants. (**A**) Tobacco plantlets were grown under sterile conditions on agar containing [U-^13^C_6_]glucose and unlabeled glucose. (**B**) ^13^CO_2_ pulse/chase experiments with whole plants of tobacco [34]. The resulting isotopologue profiles (mixtures) of nicotine are indicated on top of the respective experiments. The bars indicate multiple ^13^C-isotopologues detected by NMR in the isotopologue mixtures. Filled circles indicate single ^13^C-labeled isotopologues. The numbers indicate the mol% fractions of the respective isotopologues.

Studies on the biosynthesis of nicotine in tobacco plants have been conducted in parallel using either [U-^13^C_6_]glucose or ^13^CO_2_ as a tracer and can serve to illustrate the similarities, as well as the differences of the technologies [17,34]. Figure 5 shows labeling patterns obtained in independent experiments with (i) a tobacco plant grown aseptically on a sterile agarose matrix containing a mixture of unlabeled glucose and [U-^13^C_6_]glucose as tracer and (ii) a tobacco plant that was subjected to a pulse-chase experiment with ^13^CO_2_. Specifically, the latter plant was grown for 5 h in synthetic atmosphere containing 600 ppm ^13^CO_2_ and was then kept in the greenhouse for 10 days. In both experiments, nicotine was isolated, and its ^13^C labeling pattern was determined by NMR. In this and subsequent Figures, ensembles of two or more contiguously-labeled ^13^C blocks are indicated by bars. Most notably, the labeling patterns of nicotine from the pulse-chase experiment with ^13^CO_2_ and with [U-^13^C_6_]glucose, respectively, are closely similar. This adds additional support to the concept that ^13^CO_2_ pulse labeling is conducive to a ^13^C distribution of the central intermediary carbohydrate pools that resembles that obtained by labeling with universally ^13^C-labeled glucose.

The isotopologue compositions of nicotine from both experiments supported its well-known formation via nicotinic acid that is biosynthesized from aspartate and glyceraldehyde phosphate. The pyrrolidine ring is obtained from glutamate as reflected by the presence of two ^13^C_2_ units in the five-membered ring. For details, the reader is directed to the original paper [34]. In conclusion, the pilot experiment on nicotine biosynthesis provided strong evidence for the validity of the ^13^CO_2_ approach.

### 8.2. Hermidin

**Figure 6:**
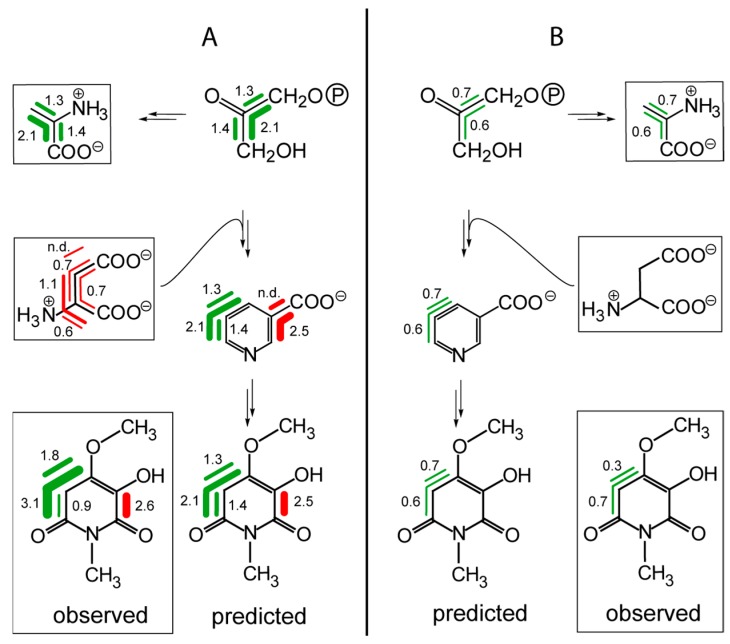
Comparison of the isotopologue profiles in hermidin from *Mercurialis annua*. (**A**) Data from the ^13^CO_2_ experiment. (**B**) Data from a labeling experiment using [U-^13^C_6_]glucose. Experimentally-observed labeling patterns are shown in boxes. The bars indicate multiple ^13^C-isotopologues detected by NMR in the isotopologue mixtures. The colors indicate molecular segments that are biosynthetically equivalent. The numbers indicate the molar amounts (mol%) of the respective isotopologues.

Parallel studies using either [U-^13^C_6_]glucose or ^13^CO_2_ as the tracer have also been conducted with hermidin, an alkaloid that is produced by the wide-spread weed, *Mercurialis annua* [38]. The similar labeling patterns obtained by the different methods are illustrated in Figure 6. Notably, the ^13^CO_2_ experiment provides higher levels of ^13^C labeling, and, as a consequence thereof, reveals substantially more labeling detail. As in the case of nicotine, the data document the incorporation of a three-carbon motif into the heterocyclic ring of hermidin. In parallel to the biosynthesis of nicotine, this suggests a biosynthetic pathway using dihydroxyacetone phosphate and aspartate as starting units. Furthermore, in parallel with the studies on nicotine biosynthesis, nicotinic acid appears as a likely intermediate in the biosynthesis of hermidin (Figure 6).

### 8.3. Nudicaulin

**Figure 7:**
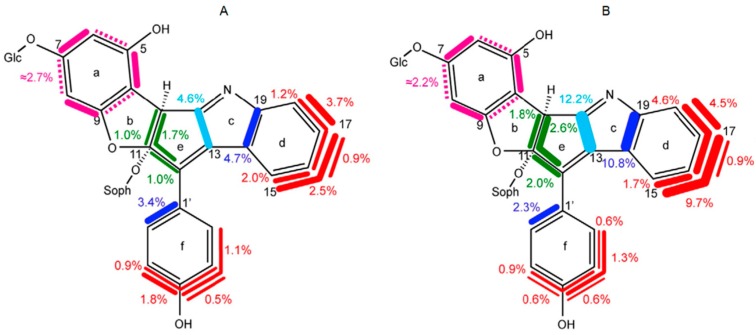
Comparison of isotopologue profiles in nudicaulin from *Papaver nudicaule* [26,27]. (**A**) From a labeling experiment with ^13^CO_2_. (**B**) From a labeling experiment with [U-^13^C_6_]glucose. For more details, see the legend to Figure 6.

Recent work has also established a closely similar labeling pattern for the flower pigment nudicaulin in parallel work with [U-^13^C_6_]glucose and ^13^CO_2_ (Figure 7) [39,40]. The biosynthetic implications of these data are discussed in the subsequent Section 11.

## 9. Biosynthesis of Terpenes Studied by ^13^CO_2_ Experiments

Terpenes are the largest family of known natural products, with at least 30,000 documented members. They participate in an almost unlimited variety of physiological processes [41]. To give just a few examples, terpenes act as components of biomembranes, pollinator attractants, dyes, hormones, vitamins, predator repellents and toxins. Numerous terpenes are major drugs serving as vitamins, hormones or cytostatic agents. On the other hand, enzymes involved in terpenoid biosynthesis and metabolism are very important drug targets in humans, most notably for the control of cholesterol biosynthesis. The remarkably large number of Nobel prizes that have been awarded for work related to terpene chemistry and biochemistry provides ample testimony of the relevance of terpene research.

Irrespective of the enormous structural complexity of the terpene family, all terpenes are biosynthesized from only two simple five-carbon compounds, namely isopentenyl diphosphate (IPP) and dimethylallyl diphosphate (DMAPP) (for a review, see [42]). Quite often, the assembly pattern, even of structurally-complex terpenes, from branched five-carbon motifs can be guessed using the “isoprenoid rule” that had been formulated by Ruziczka around the 1940s (in a comprehensive article published in 1953 in Experientia [43], Ruzicka singlehandedly presented the isoprenoid dissections for a wide variety of terpenes).

Pioneering work by Lynen, Bloch and coworkers elucidated the biosynthesis of the universal isoprenoid precursor, IPP and DMAPP, from acetyl-CoA via mevalonate (reviewed in [44]). A spinoff from this work was the introduction of blockbuster drugs inhibiting the biosynthesis of cholesterol by inhibition of a mevalonate pathway enzyme, hydroxymethyl-glutaryl-CoA reductase. Despite some inconvenient anomalies, the question of isoprenoid biosynthesis appeared settled.

Unexpectedly, the 1990s brought the discovery of a second major pathway affording IPP and DMAPP from glyceraldehyde phosphate and pyruvate via 1-deoxyxylulose 5-phosphate (DXP) and 2C-methylerythritol 4-phosphate (MEP) [45,46]. Importantly, the biosynthetic pathways of different plant terpenes take their respective isoprenoid precursors either from the mevalonate or the non-mevalonate pathway (but crosstalk between the pathways is also possible; reviewed in [42,47]).

**Figure 8:**
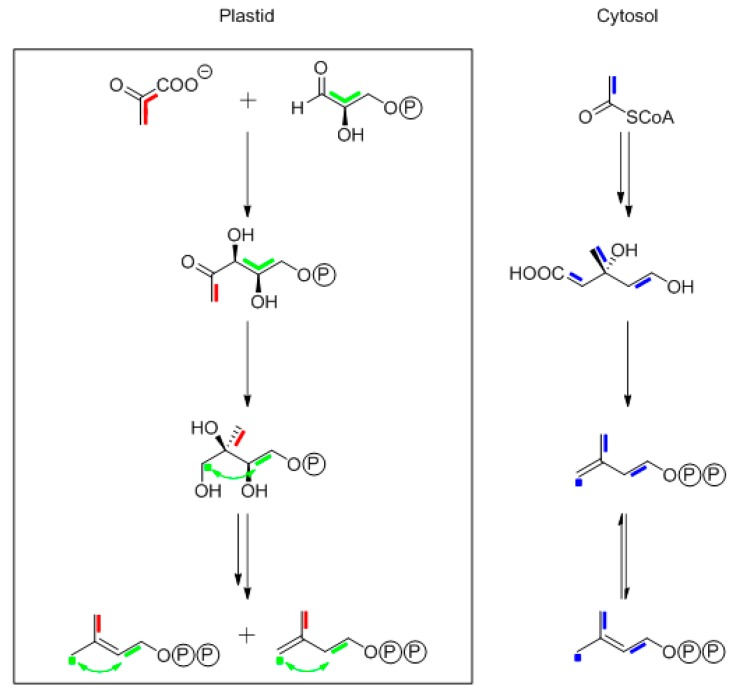
Biosynthesis of the terpene precursors, isopentenyl diphosphate (IPP) and dimethylallyl diphosphate (DMAPP), via the non-mevalonate (MEP) pathway in the plastidic compartment (in the box) of a plant cell or via the mevalonate pathway in the cytosolic compartment. Biosynthetic motifs derived from pyruvate and glyceraldehyde phosphate (GAP) are indicated by red and green colors, respectively. The green arrows indicate that the three-carbon connectivity of the original GAP precursor becomes modified by a skeletal rearrangement during the biosynthetic process. Biosynthetic units deriving from acetyl-CoA in the mevalonate route are indicated by blue colors.

In order to determine the biosynthetic origin of the isoprenoid building blocks of a given plant terpene, it is essential that the mevalonate pathway uses three acetyl-CoA precursor molecules, whereas the non-mevalonate pathway uses only two precursor molecules, i.e., pyruvate and glyceraldehyde 3-phosphate, to assemble an isoprenoid precursor molecule. That implies that the mevalonate pathway can contribute pairs of ^13^C atoms, but not ^13^C triples, whereas the non-mevalonate pathway contributes ^13^C triples (from the glyceraldehyde 3-phosphate precursor), as well as ^13^C pairs (Figure 8). It turns out that the ^13^CO_2_ pulse-chase experiment is perfectly suited to distinguish between these alternatives, as will become obvious as we proceed to specific examples.

The two different isoprenoid biosynthesis pathways are spatially separated inside plant cells, with the mevalonate pathway operating in the cytoplasmic compartment and the non-mevalonate pathway inside chloroplasts (Figure 8). To make matters more complicated, however, the isoprenoid precursors for different steps in the biosynthesis of complex terpenes can be contributed from different sources. Hence, it is of interest to specifically assess the origin of each five-carbon unit of a complex terpene. For that purpose, it is of course advantageous to be able to work at the level of intact, unperturbed plants, rather than any cellular in vitro system, which might be prone to artifacts. As shown by the subsequent examples, pulse-chase experiments with ^13^CO_2_ are perfectly suited to distinguish between the {2+2+1} pattern of mevalonate origin and the {2+3} pattern of non-mevalonate origin. Indeed, short-term ^13^CO_2_ labeling experiments with *Arabidopsis thaliana* showed that the label from ^13^CO_2_ is rapidly (within minutes) assimilated into intermediates of the non-mevalonate pathway [48].

Figure 8 illustrates possible outcomes of a thought experiment where a single molecule of [U-^13^C_3_]glyceraldehyde phosphate is contributed to an isoprenoid diphosphate unit via the shortest possible path (i.e., without reshuffling in any metabolic cycles) and in the presence of unlabeled metabolites that can be used as cosubstrates. By way of the non-mevalonate pathway, the ^13^C-labeled glyceraldehyde phosphate could afford an isoprenoid containing a pair of directly-connected carbon atoms, plus a ^13^C-labeled methyl group in DMAPP or a ^13^C-labeled methylene group in IPP, that are not directly connected to the pair, as a consequence of the skeletal rearrangement that is engineered by methylerythritol phosphate synthase. Alternatively, [U-^13^C_3_]glyceraldehyde 3-phosphate could be converted to [U-^13^C_3_]pyruvate, whose utilization for isoprenoid formation would afford a product carrying a directly-linked pair of ^13^C atoms. Notably, C-1 of pyruvate is lost as CO_2_ during the biosynthetic process (cf. Figure 8).

On the other hand, for utilization via the mevalonate pathway, the [U-^13^C_3_]glyceraldehyde phosphate molecule would have to be converted, via [U-^13^C_3_]pyruvate, into [1,2-^13^C_2_]acetyl-CoA. Since three molecules of acetyl-CoA fuel the mevalonate pathway, a single ^13^C-labeled acetyl-CoA molecule could afford two different isoprenoid isotopologues carrying directly-linked pairs of ^13^C atoms. Moreover, a double-labeled acetate unit could yield an isoprenoid isotopologue carrying a ^13^C-labeled methyl or methylene group.

Remarkably, the overall isotopologue patterns obtained via the two different pathways in our thought experiment look remarkably similar insofar as the ^13^C pairs have the same locations. Most importantly, however, only the non-mevalonate pathway can contribute three ^13^C atoms from a single precursor molecule, whereas the mevalonate pathway can at most contribute pairs of ^13^C atoms. This is the single, crucial criterion to be used for the differentiation of mevalonate versus non-mevalonate origin to be used in tracer experiments starting either with exogenously-labeled [U-^13^C_6_]glucose or ensembles of multiply-labeled carbohydrates generated in situ by photosynthesis from ^13^CO_2_. The following sections illustrate the practical application of this cutting edge criterion.

### 9.1. Thymol

**Figure 9:**
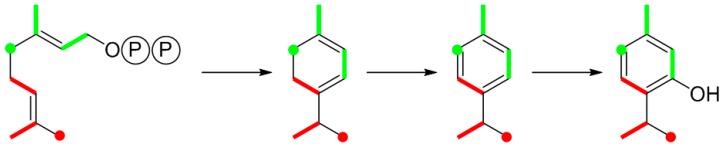
Biosynthesis of thymol from geranyl diphosphate that is formed from DMAPP (red motif) and IPP (green motif).

Thymol is a simple monoterpene. Anyone who is marginally familiar with the “isoprene rule” of terpene biosynthesis can easily execute the dissection of thymol into the two precursor isoprenoids that yield the carbon skeleton (Figure 9). The question is, however, whether these monomers arise by the {2+3} or the {2+2+1} scheme via the non-mevalonate or mevalonate pathway, respectively.

Evidence for a {2+3} origin of any isoprenoid by NMR should be easy enough, except that the three-carbon unit is disrupted by the skeletal rearrangement engineered by methylerythritol phosphate synthase (IspC) catalyzing the first committed step of the non-mevalonate pathway.

**Figure 10:**
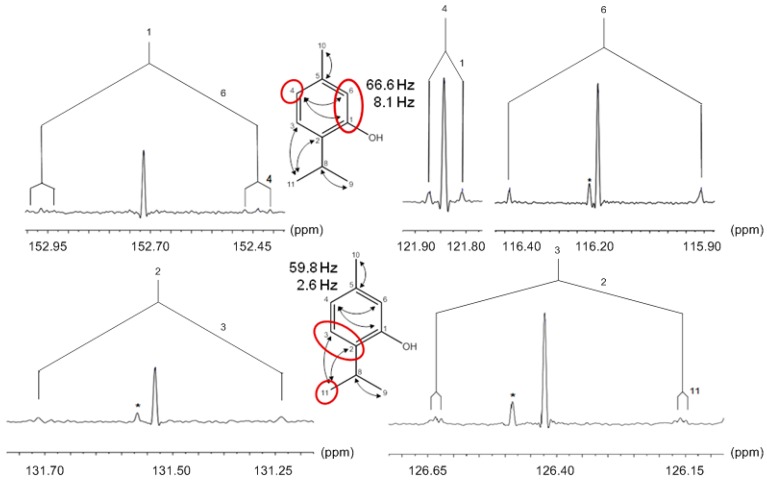
^13^C-NMR signals of thymol isolated from of *Thymus transcaucasicus* grown under an atmosphere containing ^13^CO_2_. ^13^C-Couplings via one-bond or multiple bonds are indicated with the corresponding coupling constants. Carbon atoms derived from the GAP precursor via the non-mevalonate pathway are highlighted in red. * indicates signals due to impurities.

In a ^13^CO_2_ pulse-chase experiment with *Thymus transcaucasicus*, biosynthetic thymol was analyzed exhaustively by 1D and advanced 2D NMR techniques, as well as mass spectrometry [49]. A battery of simple, as well as advanced NMR tools uniformly confirmed the presence of fragmented ^13^C triples in the monoterpene. The technical challenge is the necessity to diagnose the three-carbon fragment via long-range coupling with its characteristically small ^13^C-^13^C coupling constants. To this aim, harnessing advanced 2D-NMR techniques were used [36]. However, as shown in Figure 10, even a humble 1D ^13^C experiment can add evidence by documenting simultaneous coupling of C-1 to the directly-connected carbon atom C-6 (J, 66.6 Hz) and to the more remote carbon C-4 (J, 8.1 Hz). Similarly, a ^13^C-triple could also be detected for C-3/C-2/C-11 in the other isoprenoid unit (Figure 10). In conclusion, the isotopologue profiles demonstrated for the first time the {2+3} origin of both C_5_-precursors in the monoterpene under physiological conditions.

### 9.2. Artemisinin

Artemisinin and its derivatives are at present the most important drugs for the treatment and prevention of malaria, which is rampant in large parts of the world and claims more than 600,000 lives per year. Although synthetic artemisinin analogs are now available, the fight against malaria depends, more than anything else, on natural products from *Artemisia* plants. Notably, the Nobel price has been awarded in 2015 to Youyou Tu, at the age of 85 years, for her contribution to the discovery of the drug [50].

**Figure 11:**
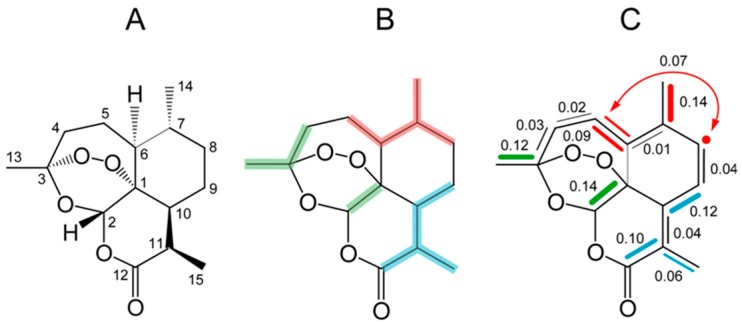
Studies on the biosynthesis of the sesquiterpene artemisinin. (**A**) Structure and carbon numbering of artemisinin. (**B**) Isoprene dissections of artemisinin indicated by the different colors. (**C**) ^13^C isotopologue composition of artemisinin from the experiment with ^13^CO_2_. Multiple ^13^C-labeled isotopologues are indicated by colored bars, single-labeled isotopologues by filled circles. The arrow indicates a ^13^C-triple detected by long-range ^13^C–^13^C couplings in the biosynthetic sample. The numbers indicate ^13^C enrichments in mol%.

Artemisinin is a sesquiterpene assembled from three isoprenoid precursor moieties via farnesyl diphosphate. The connectivity of the isoprenoid moiety shown in green in Figure 11 is disrupted at a later biosynthetic stage by oxidative cleavage of a carbon-carbon bond of a bicyclic intermediate.

Improving *Artemisia* cultivars by breeding and/or genetic engineering could help to guarantee a sufficient supply of artemisinin-based antimalarials. Understanding the biosynthetic origin of each of the three isoprenoid moieties of the farnesyl diphosphate precursor could be of practical interest in that context.

Surprisingly, the retrodiction of the building blocks in a ^13^CO_2_ pulse-chase experiment documented the inclusion of a three-carbon moiety for the middle IPP moiety (Figure 11C), whereas the DMAPP starter molecule and the terminal IPP moiety of the geranyl diphosphate precursor showed no evidence of ^13^C triples [51]. Hence, the middle isoprenoid precursor must have been generated by the non-mevalonate pathway inside chloroplasts, whereas both flanking precursors could arise via the mevalonate pathway inside the cytoplasmic compartment. Such a scenario would necessitate the transfer of DMAPP from the cytoplasmic compartment to the plastid stroma, where it could react with an IPP moiety derived via the non-mevalonate pathway. In a consecutive step, the resulting geranyl diphosphate would have to be re-exported to the cytoplasmic compartment for conversion to farnesyl diphosphate by the addition of an IPP unit of mevalonate origin. Whereas this hypothesis may need additional confirmation, the findings illustrate how the pulse-chase experiment with ^13^CO_2_ can also provide insights into the transport of intermediates between the compartments of a plant cell.

### 9.3. Ginsenosides

A plethora of putative health benefits has been attributed to the ginseng plant, and ginseng products are estimated to have a market value of about two billion US$ per year worldwide [52]. Among the numerous secondary products in ginseng extracts, triterpene type ginsenosides are dominant and are believed to be the main contributors to the claimed health benefits.

**Figure 12:**
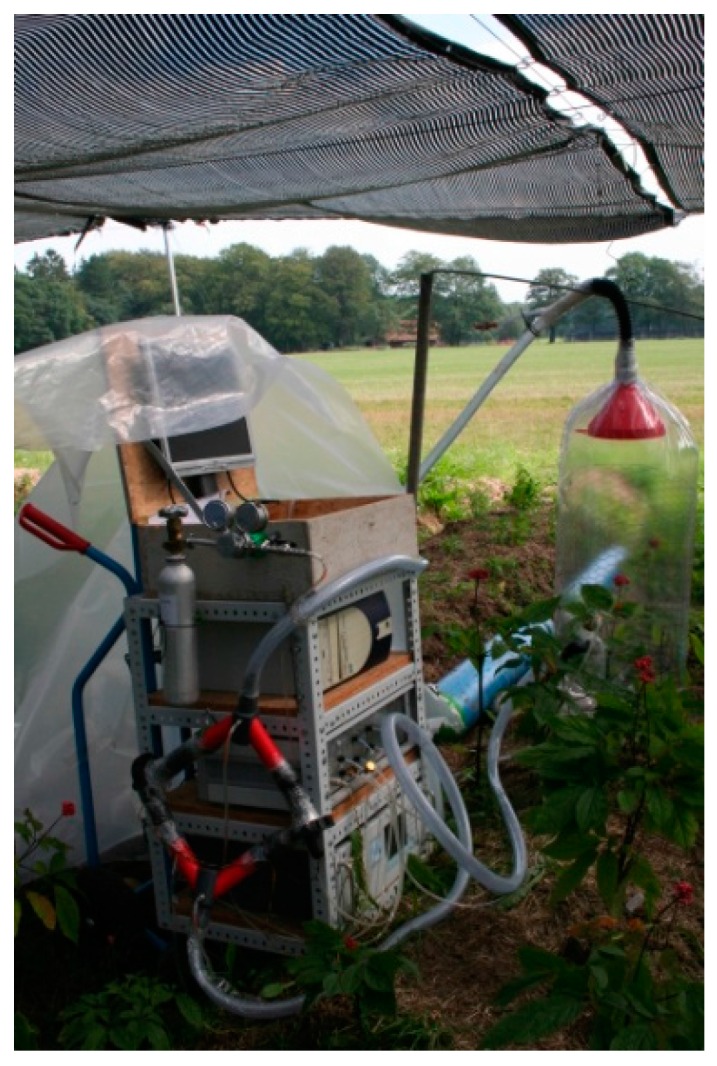
Transportable facility to label plants in the field with ^13^CO_2_. The specific example shows an experiment with *Panax ginseng* plants aimed to elucidate the biosynthesis of ginsenosides.

The world-wide ginseng supply is based on harvesting plants aged 4–8 years. Advantageously, the ^13^CO_2_ pulse-chase method can be applied under field conditions and even to plants of considerable size; thus, a six-year old ginseng plant could be used for the study of triterpene-type ginsenosides (Figure 12) [53].

**Figure 13:**
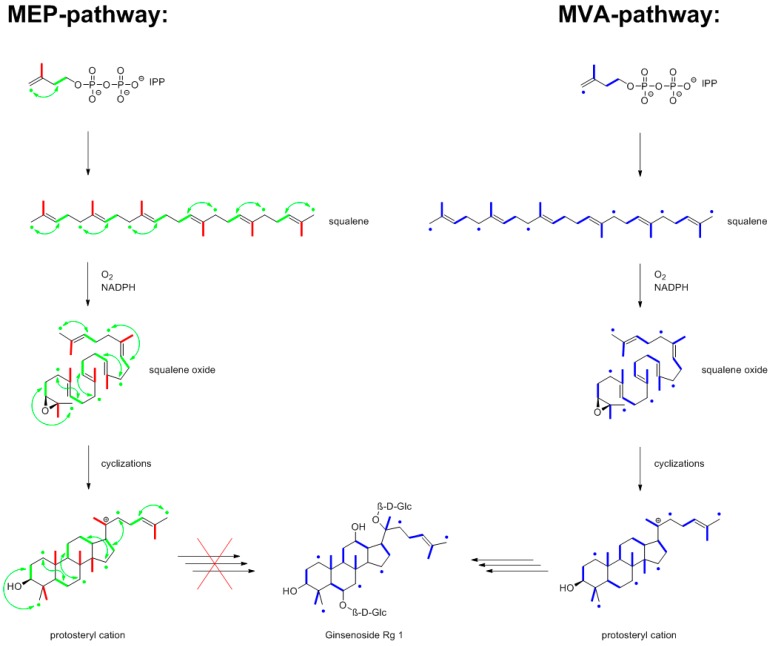
Biosynthesis of the ginsenoside Rg1 from ^13^CO_2_-labeled plants of *P. ginseng*. The bold bonds indicate adjacent ^13^C-atoms from [1,2-^13^C_2_]acetyl-CoA (colored in blue) via the mevalonate (MVA) pathway or from [U-^13^C_3_]pyruvate (colored in red) and [U-^13^C_3_]GAP (colored in green) via the MEP pathway. The observed labeling profile of Rg1 (in the middle) was perfectly in line with the MVA prediction, but at odds with the MEP prediction.

Specifically, a pulse period of 7 h followed by a chase period of eight days was applied under field conditions. The ginsenosides Rg1 and Rb1 were isolated from the roots. The small, but significant coupling satellites detected in the ^13^C-NMR spectrum gave evidence for ^13^C_2_-units in the molecules indicating the mevalonate origin of the triterpenes under study. No evidence whatsoever was obtained for the incorporation of ^13^C triples into ginsenosides. Hence, a {2+3} pattern appears most unlikely, and ginsenosides can be attributed to the mevalonate pathway. Moreover, the observed labeling patterns were in perfect agreement with a chair-chair-chair-boat conformation of the (S)-2,3-oxidosqualene precursor entering the cyclization process via the protosteryl cation (Figure 13).

### 9.4. Lupeol

**Figure 14:**
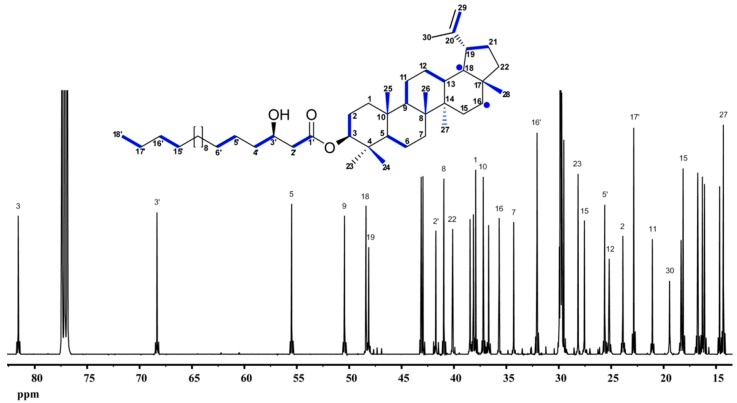
^13^C-NMR analysis of lupeol-3-(3′-R-hydroxy)-stearate from *Pentalinon andrieuxii* labeled with ^13^CO_2_. Many of the NMR signals display pairs of ^13^C-satellites. The corresponding ^13^C_2_-units in the biosynthetic compound are indicated by blue bars. A pair of ^13^C-atoms that becomes disrupted during the cyclization process is shown by blue circles (cf. also Figure 15).

Lupeol is a triterpene alcohol that has been obtained from numerous plants, with occasionally high abundance. Whereas the later stages of its biosynthesis have been elucidated in some detail, the origin of its basic isoprenoid building blocks, i.e., IPP and DMAPP, had not been established. In many cases, conjugates of lupeol and fatty acids can be found in plants. As an example, lupeol-3-(3′-R-hydroxy)-stearate is a major compound in the Mexican medicinal plant, *Pentalinon andrieuxii*. The assumed polyketide origin of the long-chain β-hydroxycarboxylic acid from acetate moieties via acetyl-CoA provides an elegant test for the power of the ^13^CO_2_ pulse-chase experiment [54]. Perfectly in line with the prediction, each observed carbon atom of the acyl moiety is exclusively related by ^13^C-^13^C coupling to a single adjacent carbon atom, thus unequivocally identifying the biosynthetic acetate building blocks (Figure 14).

**Figure 15:**
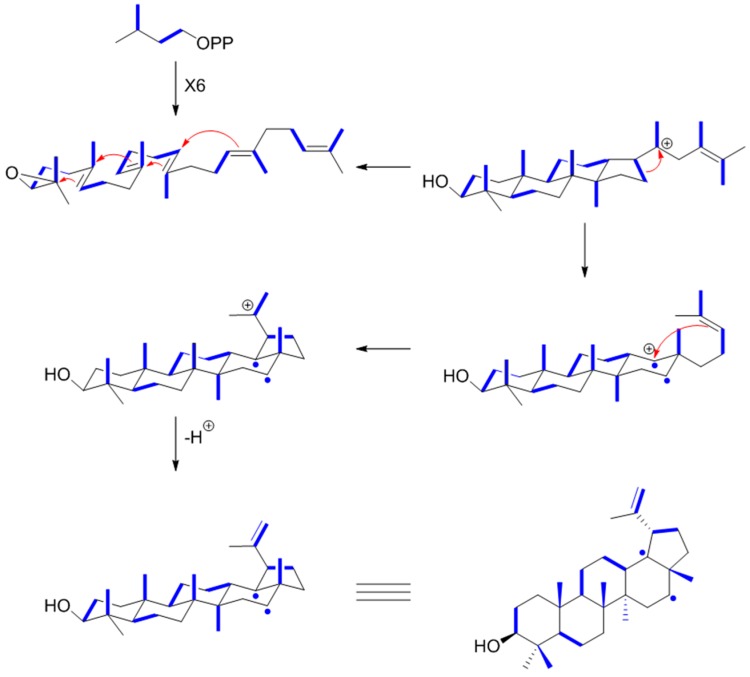
Biosynthetic steps in the formation of lupeol. ^13^C_2_ units from ^13^C_2_-acetyl-CoA via the mevalonate pathway are indicated by blue bars. One pair of ^13^C atoms becomes disrupted during the cyclization process and affords the single ^13^C atoms in the structure shown by blue circles.

Likewise, the pentacyclic isoprenoid moiety shows ^13^CO_2_ pairs, but no ^13^C triples whatsoever. Hence, the isoprenoid moiety is exclusively of mevalonate origin. Notably, one of the ^13^C_2_-moieties is fractured by skeletal rearrangement in the later course of the biosynthetic pathway (Figure 15).

## 10. Polyacetylenes from *Panax ginseng*

**Figure 16:**
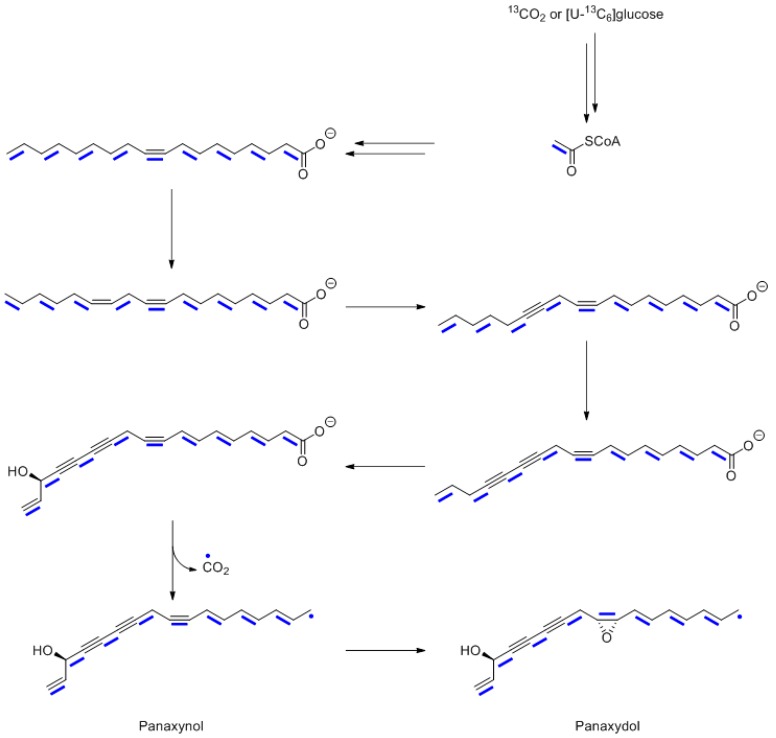
Proposed biosynthetic pathway of panaxynol and panaxydol. Adjacent ^13^C atoms detected in experiments with ^13^CO_2_ or [U-^13^C_6_]glucose are indicated by blue bars.

In close similarity to the labeling pattern of the fatty acid residue of the lupeol derivative, a strict polyketide pattern was also observed for ^13^CO_2_-labeled panaxynol and panaxydol from the plethora of secondary products formed by *Panax ginseng* [55]. Specifically, the number of ^13^C atoms that had been imported jointly does not exceed two in any case. Notably, however, the terminal methyl carbon atoms of panaxynol or panaxydol did not show any co-transfer at all, thus suggesting that one acetate moiety in the polyketide precursor has been shortened by the loss of a one-carbon unit (Figure 16).

## 11. Tracking Shikimate Derivatives as Building Blocks for Secondary Metabolites

**Figure 17:**
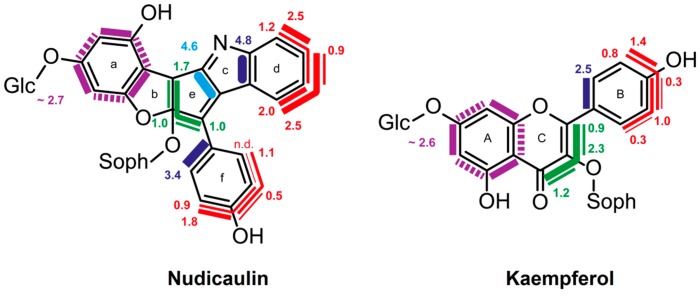
Isotopologue compositions of nudicaulin and kaempferol from ^13^CO_2_ pulse/chase experiments with *Papaver nudicaule* [26]. The bars indicate multiple ^13^C-labeled isotopologues. The numbers indicate mol% of the respective isotopologues. The colors indicate related precursor units (for details, see the text).

Figure 17 summarizes data from a ^13^CO_2_ pulse-chase experiment with *Papaver nudicaule* [39]. One of the compounds in Figure 17, nudicaulin, is responsible for the yellow color of the plant’s flowers. The other, kaempferol, is a flavonoid whose occurrence is shared with a variety of plant species.

The similarity of the rings f and B with, regard to the structure and labeling pattern are immediately obvious. Thus, these rings of both compounds can acquire contiguous blocks of up to three ^13^C atoms, notably with overlapping topology. This is easily explained if the phenol rings arise from a four-carbon precursor that can be endowed with different labeling patterns; more specifically, the four-carbon element is easily tracked to erythrose 4-phosphate, a precursor in the shikimate pathway that is the sole source for aromatic amino acid biosynthesis (see above). In both compounds shown in Figure 17, the rings b/e and C display a linker consisting of three carbon atoms shown in green. Importantly, that linker can simultaneously import up to three ^13^C atoms, thus suggesting that it represents the side chain of a tyrosine precursor, such as coumarate.

**Figure 18:**
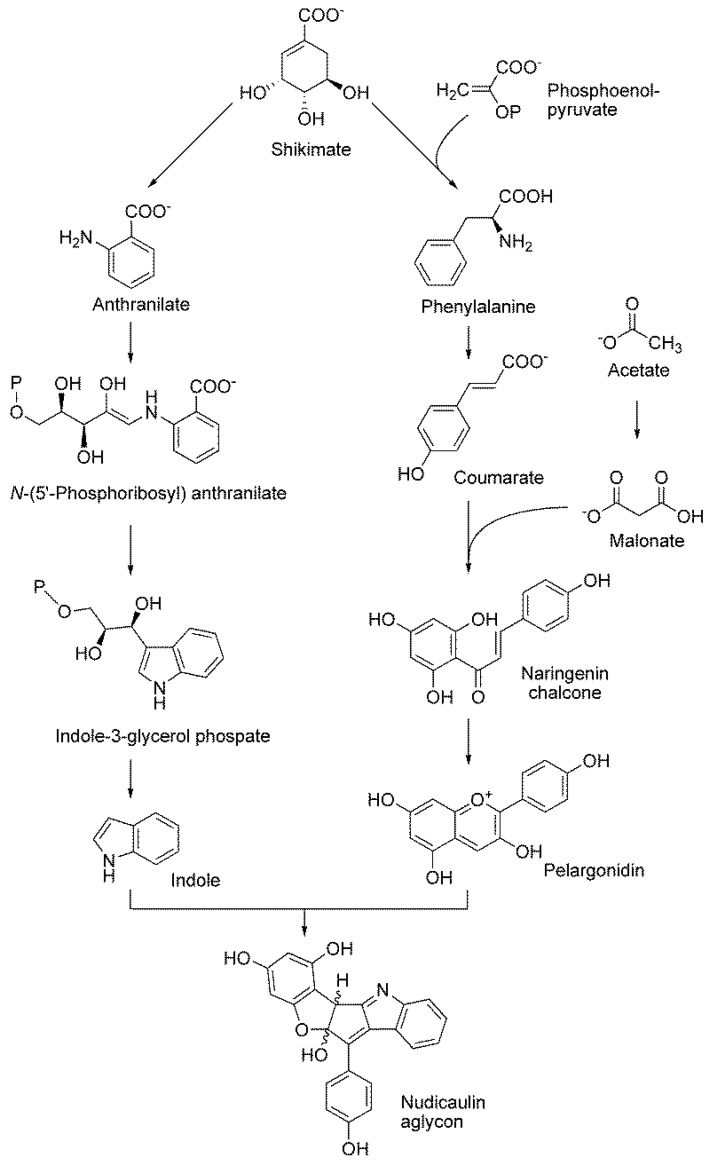
Proposed biosynthetic pathway for nudicaulin biosynthesis [40].

The labeling pattern of rings a and A may appear surprising at first sight. ^13^C-^13^C coupling is displayed for each ring carbon to each of the two adjacent ring atoms; however, no ring carbon is ever simultaneously coupled to both adjacent carbon atoms. This seeming paradox is in fact a well-known feature of ring systems of polyketide origin, as shown by the reaction sequence proposed in Figure 18. Briefly, the tyrosine precursor coumarate is extended by a sequence of ester condensations using malonyl CoA as the substrate. Ring closure affords a trihydroxyphenyl moiety that is initially free to rotate, but is subsequently immobilized by the introduction of an ether bridge that results in the fixation of two discrete labeling patterns for ring a.

Unsurprisingly, the benzenoid ring of the indole motif in nudicaulin repeats in every detail the shikimate labeling pattern that we have seen in the phenolic ring of both compounds from *P. nudicaule*. The yellow pigment could arise by condensation of the tryptophan precursor, indole, with the shikimate/polyketide-type intermediate pelargonidin via naringenin chalcone (Figure 18) [40].

## 12. The Role of the Time Coordinate in Work with ^13^CO_2_

Numerous plant physiology studies have used ^13^CO_2_ to analyze the kinetics of the transfer of primary photosynthate into different metabolic compartments. Not surprisingly, time is the most important experimental parameter in work of this type that is not discussed in detail in the present review.

On the other hand, the work on biosynthesis pathways discussed in the present review is entirely focused on the identification of biosynthetic building blocks, based on the concept that the carbon skeletons of secondary metabolites are mosaic structures that are assembled from “Lego^©^” blocks extracted from primary metabolite. Technically, that implicates the detection of covalently-linked ^13^C entities inside the target molecule structures by ^13^C-^13^C coupling via NMR and by mass spectrometric detection of multiply ^13^C-labeled isotopologues. In other words, the required information resides in isotopologue space, as opposed to ime space.

Closer analysis, however, reveals easily that the time coordinate is also relevant in biosynthetic studies. In fact, the entire concept is based on the assembly of an inventory of multiply ^13^C-labeled metabolites by a dynamic process, namely the formation of highly ^13^C-labeled photosynthate in a ^13^CO_2_-containing atmosphere and its subsequent dispersal, by mixing with pools of unlabeled photosynthate. Whereas the isotopologue distribution of biomatter is normally very close to stochastic, a massive disequilibrium can be generated by a dynamic process, namely the ^13^CO_2_ labeling pulse. The subsequent relaxation period is conducive to mixing of metabolites from highly different compartments of isotopologue space, namely the photosynthate from ^13^CO_2_ with biomass that had been deposited in the prelabeling period and with biomass that is assembled de novo during the chase period.

The chase (relaxation) period is conducive to the generation of novel isotopologues by recombination of building blocks from unlabeled and partially or completely ^13^C-labeled precursor molecules. However, the chase period does not have a tendency to reestablish a truly stochastic isotope distribution within the timescale of realistic experiments. Rather, the return to a stochastic distribution, inside a closed system, would require very long periods of metabolization and would typically have to involve multiple species, including predators of plant biomass. In terms of isotopologue entropy, the chase period develops in the direction of a metastable state, rather than a new quasi-stochastic isotopologue distribution.

A systematic optimization of pulse and chase duration has not been performed for the type of biosynthetic studies described in the first part of the article. In fact, the conditions used, with a pulse of a typical sunlight day and a chase period of several days, were essentially established serendipitously. In retrospect, it appears that the lengths of the time periods are not really critical in light of the fact that any state with a lowered isotope entropy that has been generated by the pulse period will coast toward a metastable state of isotopologue entropy during the chase period, rather than to a novel equilibrium state with an enhanced average abundance. In any case, the presently available data on biosynthetic studies using ^13^CO_2_ as a universal precursor provide ample evidence that the approach is conducive to generating information on carbon metabolism that cannot easily be obtained by other techniques.

## Figures and Tables

**Table 1:** Reconstruction of the labeling patterns of biosynthetic precursors^a^ from the ^13^C-patterns of key amino acids.

Amino Acid	Precursor^a^	Related Upstream Intermediate	Pathway
Ala	Pyruvate	Glyceraldehyde 3-Phosphate, Malate	Glycolysis, C4-dicarboxylic acid cycle, Non-mevalonate pathway
Arg	Glu	α-Ketoglutarate	Citrate cycle
Arg	CO_2_		C1-metabolism
Asp	Oxaloacetate	Phosphoenolpyruvate, CO_2_	Citrate cycle, C1-metabolism
Glu	α-Ketoglutarate	Oxaloacetate, Acetyl-CoA	Citrate cycle, Mevalonate pathway
His	5-Phosphoribosyl diphosphate	Ribulose 5-Phosphate	Pentose phosphate pathway, Calvin cycle, C1-metabolism
His	ATP	CO_2_	Purine metabolism, C1-metabolism
Ile	Thr	Asp	Citrate cycle
Ile	Pyruvate	Glyceraldehyde 3-Phosphate, Malate	Glycolysis, C4-dicarboxylic acid cycle, Non-mevalonate pathway
Leu	Pyruvate	Glyceraldehyde 3-Phosphate, Malate	Glycolysis, C4-dicarboxylic acid cycle, Non-mevalonate pathway
Leu	Acetyl-CoA	Pyruvate, Fatty acids	Mevalonate pathway, Polyketide metabolism, Fatty acid metabolism
Lys	Asp	Oxaloacetate	Citrate cycle
Phe	Erythrose 4-phosphate	Fructose 6-Phosphate, Ribulose 5-Phosphate	Chorismate pathway, Pentose phosphate pathway, Calvin cycle
Phe	Phosphoenol-pyruvate	3-Phosphoglycerate, Oxaloacetate	Chorismate pathway, Glycolysis, Calvin cycle
Pro	Glu	α-Ketoglutarate	Citrate cycle
Thr	Asp	Oxaloacetate	Citrate cycle
Trp	Erythrose 4-phosphate	Fructose 6-Phosphate, Ribulose 5-Phosphate	Chorismate pathway, Pentose phosphate pathway, Calvin cycle
Trp	Phosphoenol pyruvate	3-Phosphoglycerate, Oxaloacetate	Chorismate pathway, Glycolysis, Calvin cycle
Trp	Ser	3-Phosphoglycerate, Gly	Chorismate pathway, Glycolysis, Calvin cycle, C1-metabolism
Trp	5-Phosphoribosyl diphosphate	Ribulose 5-Phosphate	Pentose phosphate pathway, Calvin cycle
Tyr	Erythrose 4-phosphate	Fructose 6-Phosphate, Ribulose 5-Phosphate	Chorismate pathway, Pentose phosphate pathway, Calvin cycle
Tyr	Phosphoenol pyruvate	3-Phosphoglycerate, Oxaloacetate	Chorismate pathway, Glycolysis, Calvin cycle
Val	Pyruvate	Glyceraldehyde 3-Phosphate, Malate	Glycolysis, C4-dicarboxylic acid cycle, Non-mevalonate pathway

^a^ These precursors also serve as key intermediates in central metabolic pathways, and, as simple building units, in the biosynthetic pathways of complex natural products. For example, acetyl-CoA or pyruvate/glyceraldehyde phosphate (GAP) are basic precursors in terpene biosynthesis, acetyl-CoA serves in the formation of polyketides, phenylalanine in the biosynthesis of phenylpropanoids, and erythrose 4-phosphate and ribose 5-phosphate in the generation of glycosides).

## References

[1] Schönheimer R., Rittenberg D. (1935). Deuterium as an indicator in the study of intermediary metabolism. Science.

[2] Kamen M.D. (1947). Use of isotopes in biochemical research; fundamental aspects. Annu. Rev. Biochem..

[3] Calvin M., Benson A.A. (1949). The path of carbon in photosynthesis IV: The identity and sequence of the intermediates in sucrose synthesis. Science.

[4] Bothwell J.H., Griffin J.L. (2011). An introduction to biological nuclear magnetic resonance spectroscopy. Biol. Rev. Camb. Philos. Soc..

[5] Boughton B.A., Thinagaran D., Sarabia D., Bacic A., Roessner U. (2016). Mass spectrometry imaging for plant biology: A review. Phytochem. Rev..

[6] Schmidt H.L., Robins R.J., Werner R.A. (2015). Multi-factorial in vivo stable isotope fractionation: Causes, correlations, consequences and applications. Isotopes Environ. Health Stud..

[7] Kanehisa M., Sato Y., Kawashima M., Furumichi M., Tanabe M. (2016). KEGG as a reference resource for gene and protein annotation. Nucleic Acids Res..

[8] Strogatz S.H. (2001). Exploring complex networks. Nature.

[9] Schmidt S., Sunyaev S., Bork P., Dandekar T. (2003). Metabolites: A helping hand for pathway evolution?. Trends Biochem. Sci..

[10] Grubmüller S., Schauer K., Goebel W., Fuchs T.M., Eisenreich W. (2014). Analysis of carbon substrates used by *Listeria monocytogenes* during growth in J774A.1 macrophages suggests a bipartite intracellular metabolism. Front. Cell. Infect. Microbiol..

[11] Häuslein I., Manske C., Goebel W., Eisenreich W., Hilbi H. (2016). Pathway analysis using ^13^C-glycerol and other carbon tracers reveals a bipartite metabolism of *Legionella pneumophila*. Mol. Microbiol..

[12] Maeda H., Dudareva N. (2012). The shikimate pathway and aromatic amino acid biosynthesis in plants. Annu. Rev. Plant Biol..

[13] Zrenner R., Stitt M., Sonnewald U., Boldt R. (2006). Pyrimidine and purine biosynthesis and degradation in plants. Annu. Rev. Plant Biol..

[14] Haase I., Gräwert T., Illarionov B., Bacher A., Fischer M. (2014). Recent advances in riboflavin biosynthesis. Methods Mol. Biol..

[15] Hanson A.D., Gregory J.F. (2011). Folate biosynthesis, turnover, and transport in plants. Annu. Rev. Plant Biol..

[16] Mendel R.R., Leimkühler S. (2015). The biosynthesis of the molybdenum cofactors. J. Biol. Inorg. Chem..

[17] Ettenhuber C., Radykewicz T., Kofer W., Koop H.U., Bacher A., Eisenreich W. (2005). Metabolic flux analysis in complex isotopolog space. Recycling of glucose in tobacco plants. Phytochemistry.

[18] Nargund S., Misra A., Zhang X., Coleman G.D., Sriram G. (2014). Flux and reflux: Metabolite reflux in plant suspension cells and its implications for isotope-assisted metabolic flux analysis. Mol. Biosyst..

[19] Schwender J., Hebbelmann I., Heinzel N., Hildebrandt T., Rogers A., Naik D., Klapperstuck M., Braun H.P., Schreiber F., Denolf P. (2015). Quantitative multilevel analysis of central metabolism in developing oilseeds of oilseed rape during in vitro culture. Plant Physiol..

[20] Kruger N.J., Ratcliffe R.G. (2015). Fluxes through plant metabolic networks: Measurements, predictions, insights and challenges. Biochem. J..

[21] Ishihara H., Obata T., Sulpice R., Fernie A.R., Stitt M. (2015). Quantifying protein synthesis and degradation in *Arabidopsis* by dynamic ^13^CO_2_ labeling and analysis of enrichment in individual amino acids in their free pools and in protein. Plant Physiol..

[22] Gao J.Q., Gao J.J., Zhang X.W., Xu X.L., Deng Z.H., Yu F.H. (2015). Effects of waterlogging on carbon assimilate partitioning in the Zoige alpine wetlands revealed by ^13^CO_2_ pulse labeling. Sci. Rep..

[23] Epron D., Cabral O.M., Laclau J.P., Dannoura M., Packer A.P., Plain C., Battie-Laclau P., Moreira M.Z., Trivelin P.C., Bouillet J.P. (2016). In situ ^13^CO_2_ pulse labelling of field-grown eucalypt trees revealed the effects of potassium nutrition and throughfall exclusion on phloem transport of photosynthetic carbon. Tree Physiol..

[24] Dersch L.M., Beckers V., Rasch D., Melzer G., Bolten C.J., Kiep K., Becker H., Blasing O.E., Fuchs R., Ehrhardt T. (2016). High-throughput plant metabolic profiling by stable isotope labelling and combustion isotope ratio mass spectrometry: In vivo assimilation and molecular re-allocation of carbon and nitrogen in rice. Plant Physiol..

[25] Hasunuma T., Harada K., Miyazawa S., Kondo A., Fukusaki E., Miyake C. (2010). Metabolic turnover analysis by a combination of in vivo ^13^C-labelling from ^13^CO_2_ and metabolic profiling with CE-MS/MS reveals rate-limiting steps of the C3 photosynthetic pathway in *Nicotiana tabacum* leaves. J. Exp. Bot..

[26] Huege J., Sulpice R., Gibon Y., Lisec J., Koehl K., Kopka J. (2007). GC-EI-TOF-MS analysis of in vivo carbon-partitioning into soluble metabolite pools of higher plants by monitoring isotope dilution after ^13^CO_2_ labelling. Phytochemistry.

[27] Ma F., Jazmin L.J., Young J.D., Allen D.K. (2014). Isotopically nonstationary ^13^C flux analysis of changes in *Arabidopsis thaliana* leaf metabolism due to high light acclimation. Proc. Natl. Acad. Sci. USA.

[28] Hutchinson C.R., Stephen M.T., Hsia S., Carver R.A. (1976). Biosynthetic studies with ^13^CO_2_ of secondary plant metabolites. *Nicotiana* alkaloids. 1. Initial experiments. J. Am. Chem. Soc..

[29] Cegelski L., Schaefer J. (2006). NMR determination of photorespiration in intact leaves using in vivo ^13^CO_2_ labeling. J. Magn. Reson..

[30] Dirks R.C., Singh M., Potter G.S., Sobotka L.G., Schaefer J. (2012). Carbon partitioning in soybean (*Glycine max*) leaves by combined ^11^C and ^13^C labeling. New Phytol..

[31] Yu T.Y., Singh M., Matsuoka S., Patti G.J., Potter G.S., Schaefer J. (2010). Variability in C_3_-plant cell-wall biosynthesis in a high-CO_2_ atmosphere by solid-state NMR spectroscopy. J. Am. Chem. Soc..

[32] Stidham M.A., Moreland D.E., Siedow J.N. (1983). C Nuclear magnetic resonance studies of Crassulacean acid metabolism in intact leaves of *Kalanchoe tubiflora*. Plant Physiol..

[33] Bassham J.A., Benson A.A., Calvin M. (1950). The path of carbon in photosynthesis. J. Biol. Chem..

[34] Römisch-Margl W., Schramek N., Radykewicz T., Ettenhuber C., Eylert E., Huber C., Römisch-Margl L., Schwarz C., Dobner M., Demmel N. (2007). ^13^CO_2_ as a universal metabolic tracer in isotopologue perturbation experiments. Phytochemistry.

[35] Eisenreich W., Ettenhuber C., Laupitz R., Theus C., Bacher A. (2004). Isotopolog perturbation techniques for metabolic networks: Metabolic recycling of nutritional glucose in *Drosophila melanogaster*. Proc. Natl. Acad. Sci. USA.

[36] Ettenhuber C., Spielbauer G., Margl L., Hannah L.C., Gierl A., Bacher A., Genschel U., Eisenreich W. (2005). Changes in flux pattern of the central carbohydrate metabolism during kernel development in maize. Phytochemistry.

[37] Eisenreich W., Bacher A. (2007). Advances of high-resolution NMR techniques in the structural and metabolic analysis of plant biochemistry. Phytochemistry.

[38] Ostrozhenkova E., Eylert E., Schramek N., Golan-Goldhirsh A., Bacher A., Eisenreich W. (2007). Biosynthesis of the chromogen hermidin from *Mercurialis annua* L.. Phytochemistry.

[39] Tatsis E.C., Eylert E., Maddula R.K., Ostrozhenkova E., Svatos A., Eisenreich W., Schneider B. (2014). Biosynthesis of nudicaulins: A ^13^CO_2_-pulse/chase labeling study with *Papaver nudicaule*. Chembiochem.

[40] Warskulat A.C., Tatsis E.C., Dudek B., Kai M., Lorenz S., Schneider B. (2016). Unprecedented utilization of pelargonidin and indole for the biosynthesis of plant indole alkaloids. Chembiochem.

[41] Tholl D. (2015). Biosynthesis and biological functions of terpenoids in plants. Adv. Biochem. Eng. Biotechnol..

[42] Eisenreich W., Bacher A., Arigoni D., Rohdich F. (2004). Biosynthesis of isoprenoids via the non-mevalonate pathway. Cell. Mol. Life Sci..

[43] Ruzicka L. (1953). The isoprene rule and the biogenesis of terpenic compounds. Experientia.

[44] Kennedy E.P., Westheimer F.H. (1964). Nobel Laureates: Bloch and Lynen win prize in medicine and physiology. Science.

[45] Arigoni D., Sagner S., Latzel C., Eisenreich W., Bacher A., Zenk M.H. (1997). Terpenoid biosynthesis from 1-deoxy-D-xylulose in higher plants by intramolecular skeletal rearrangement. Proc. Natl. Acad. Sci. USA.

[46] Rohmer M., Knani M., Simonin P., Sutter B., Sahm H. (1993). Isoprenoid biosynthesis in bacteria: A novel pathway for the early steps leading to isopentenyl diphosphate. Biochem. J..

[47] Hemmerlin A., Harwood J.L., Bach T.J. (2012). A raison d’etre for two distinct pathways in the early steps of plant isoprenoid biosynthesis?. Prog. Lipid Res..

[48] Wright L.P., Rohwer J.M., Ghirardo A., Hammerbacher A., Ortiz-Alcaide M., Raguschke B., Schnitzler J.P., Gershenzon J., Phillips M.A. (2014). Deoxyxylulose 5-phosphate synthase controls flux through the methylerythritol 4-phosphate pathway in *Arabidopsis*. Plant Physiol..

[49] Kutzner E., Manukyan A., Eisenreich W. (2014). Profiling of terpene metabolism in ^13^CO_2_-labelled *Thymus transcaucasicus*. Metabolomics.

[50] Kong L.Y., Tan R.X. (2015). Artemisinin, a miracle of traditional Chinese medicine. Nat. Prod. Rep..

[51] Schramek N., Wang H., Römisch-Margl W., Keil B., Radykewicz T., Winzenhörlein B., Beerhues L., Bacher A., Rohdich F., Gershenzon J. (2010). Artemisinin biosynthesis in growing plants of *Artemisia annua*. A ^13^CO_2_ study. Phytochemistry.

[52] Baeg I.H., So S.H. (2013). The world ginseng market and the ginseng (Korea). J. Ginseng Res..

[53] Schramek N., Huber C., Schmidt S., Dvorski S.E., Knispel N., Ostrozhenkova E., Pena-Rodriguez L.M., Cusido R.M., Wischmann G., Eisenreich W. (2014). Biosynthesis of ginsenosides in field-grown *Panax ginseng*. JSM Biotechnol. Bioeng..

[54] Pena-Rodriguez L.M., Yam-Puc A., Knispel N., Schramek N., Huber C., Grassberger C., Ramirez-Torres F.G., Escalante-Erosa F., Garcia-Sosa K., Hiebert-Giesbrecht M.R. (2014). Isotopologue profiling of triterpene formation under physiological conditions. Biosynthesis of lupeol-3-(3′-*R*-hydroxy)-stearate in *Pentalinon andrieuxii*. J. Org. Chem..

[55] Knispel N., Ostrozhenkova E., Schramek N., Huber C., Pena-Rodriguez L.M., Bonfill M., Palazon J., Wischmann G., Cusido R.M., Eisenreich W. (2013). Biosynthesis of panaxynol and panaxydol in *Panax ginseng*. Molecules.

